# Effectiveness and safety of thread embedding acupuncture for facial nerve palsy sequelae: a protocol for a multicenter, assessor-blinded, randomized, controlled clinical trial

**DOI:** 10.3389/fneur.2025.1668688

**Published:** 2025-11-12

**Authors:** Jung-Hyun Kim, Bonhyuk Goo, Dongmin Lee, Tae-Yoon Kim, Jun-Gyu Park, Eunseok Kim, Sang-Soo Nam

**Affiliations:** 1 Department of Acupuncture and Moxibustion, Kyung Hee University Hospital at Gangdong, Seoul, Republic of Korea; 2 Department of Clinical Korean Medicine, Graduate School, Kyung Hee University, Seoul, Republic of Korea; 3 Department of Clinical Korean Traditional Medicine, Jaseng Medical Foundation, Seoul, Republic of Korea; 4 Division of Clinical Medicine, School of Korean Medicine, Pusan National University, Yangsan, Republic of Korea; 5 Department of Acupuncture and Moxibustion Medicine, Pusan National University Korean Medicine Hospital, Yangsan, Republic of Korea; 6 Department of Acupuncture and Moxibustion, Kyung Hee University College of Korean Medicine, Kyung Hee University Hospital at Gangdong, Seoul, Republic of Korea

**Keywords:** facial nerve palsy, thread embedding acupuncture, Facial Disability Index, facial stiffness, synkinesis, randomized controlled trial

## Abstract

**Introduction:**

Facial nerve palsy can result in various sequelae. Recent reviews and clinical studies reported that thread-embedding acupuncture is a safe treatment and can improve facial nerve palsy sequelae. This trial aims to confirm the effectiveness and safety of thread-embedding acupuncture for patients with facial nerve palsy sequelae.

**Methods:**

A multicenter, randomized, assessor-blinded clinical trial will be conducted. One hundred forty-two participants will be recruited by eligibility criteria and be randomly divided into two groups. The intervention group will receive thread-embedding acupuncture in addition to electroacupuncture, while the control group will be given only electroacupuncture. The primary outcome is a change in the physical function score of the Facial Disability Index. The secondary outcome is change in Sunnybrook Facial Grading System, Social/Wellbeing function score of Facial Disability Index, Synkinesis Assessment Questionnaire, Numeric Rating Scale of stiffness by facial region, Patient Global Impression of Change, Euro-Qol 5-Dimension 5-level, Economic evaluation questionnaire and adverse events. Assessment points include before treatment (Week 1), after treatment completion (Week 6), 2 weeks post-treatment completion (Week 8, Primary endpoint), and 10 weeks post-treatment completion (Week 16).

**Conclusion:**

This randomized controlled trial will assess the effectiveness and safety of thread-embedding acupuncture treatment alongside electroacupuncture for facial nerve palsy, using proper sample sizes and rigorous methodologies to address previous research limitations.

**Clinical trial registration:**

https://cris.nih.go.kr/cris/search/detailSearch.do?seq=25846&status=5&seq_group=24726&search_page=M, identifier KCT0008551.

## 1 Introduction

Facial nerve palsy (FNP) is an acute palsy that appears on the face, with an incidence of approximately 20–25 cases per 100,000 people annually. It involves partial or complete loss of facial movement, accompanied by symptoms such as taste abnormalities in the front part of the tongue, hypersensitivity of hearing, tinnitus, and postauricular pain. Although most cases of FNP recover usually, some may result in sequelae (1). The progress of FNP shows that approximately 71% of patients can fully recover without treatment within 6 months; however, 29% may experience residual sequelae (2). The sequelae of facial palsy typically manifest about 3–6 months after onset and can include synkinesis, contracture, spasm, and crocodile tears syndrome. The prevalence of specific sequelae symptoms is as follows: contracture in 17%, synkinesis in 16%, crocodile tear syndrome in 2%, and ocular dryness in 2% (3).

Long-term sequelae of FNP can make it difficult for patients to express themselves through facial gestures, leading to discomfort in daily life and professional activities due to facial deformities, stiffness, and irregular movements. Furthermore, these facial changes can result in a decline in self-confidence and self-esteem, causing patients to hesitate to engage with others and encounter difficulties in social activities (4).

While the acute phase treatment of facial palsy is well standardized, only two approaches, nerve transplantation, and botulinum toxin treatment, are known for treating post-sequelae. Furthermore, due to limited evidence supporting these methods, patients are only offered restricted options (5). Consequently, there has been a growing interest in non-surgical therapies using complementary and alternative medicine (6). Recently, thread-embedding acupuncture (TEA) has been utilized in treating FNP. TEA is a method in which an external material is embedded within the acupoint. This material can be used to continuously stimulate the meridian and consequently treat diseases (6). According to a systematic review on the safety of TEA reported in 2021, an examination of 45 randomized controlled trials and 16 case reports revealed that TEA is a very safe treatment. Notably, no serious adverse events occurred when using polydioxanone thread material predominantly utilized in Korea (7). Additionally, a review of clinical reports from Korea on facial thread embedding treatment revealed that, apart from mild cases of bruising, there were no significant side effects, making it a relatively safe treatment (8).

In 2020, we reported the results of a clinical trial comparing TEA with sham TEA for the FNP sequelae. Although we could explore the clinical potential of TEA for the FNP sequelae, since this was the first clinical trial conducted, several limitations were found in the study design (9). To overcome these limitations, this clinical trial aims to confirm the effectiveness and safety of the FNP sequelae depending on whether or not TEA is performed based on full-scale design, appropriate sample calculation, and comparison similar to clinical situations.

## 2 Methods

### 2.1 Trial design

This protocol was designed according to the standard protocol items: recommendations for interventional trials (SPIRIT) checklist and Standards for Reporting Interventions in Clinical Trials of Acupuncture (STRICTA) 2010 checklist of information to include when reporting interventions in a clinical trial of thread embedding. The study protocol (FTEA-2023-01, version: 1.5) was approved by the Institutional Review Board (IRB) of the Kyung Hee University Oriental Hospital at Gangdong (Approval Number: KHNMCOH IRB 2022-09-001) and the Pusan National University Korean Medicine Hospital (Approval Number: PNUKHIRB 2022-09-003). Additionally, this study was registered in the Clinical Research Information Service (KCT0008551). If there are any changes in the study design, the Ethics Committee will be immediately informed.

**Figure 1:**
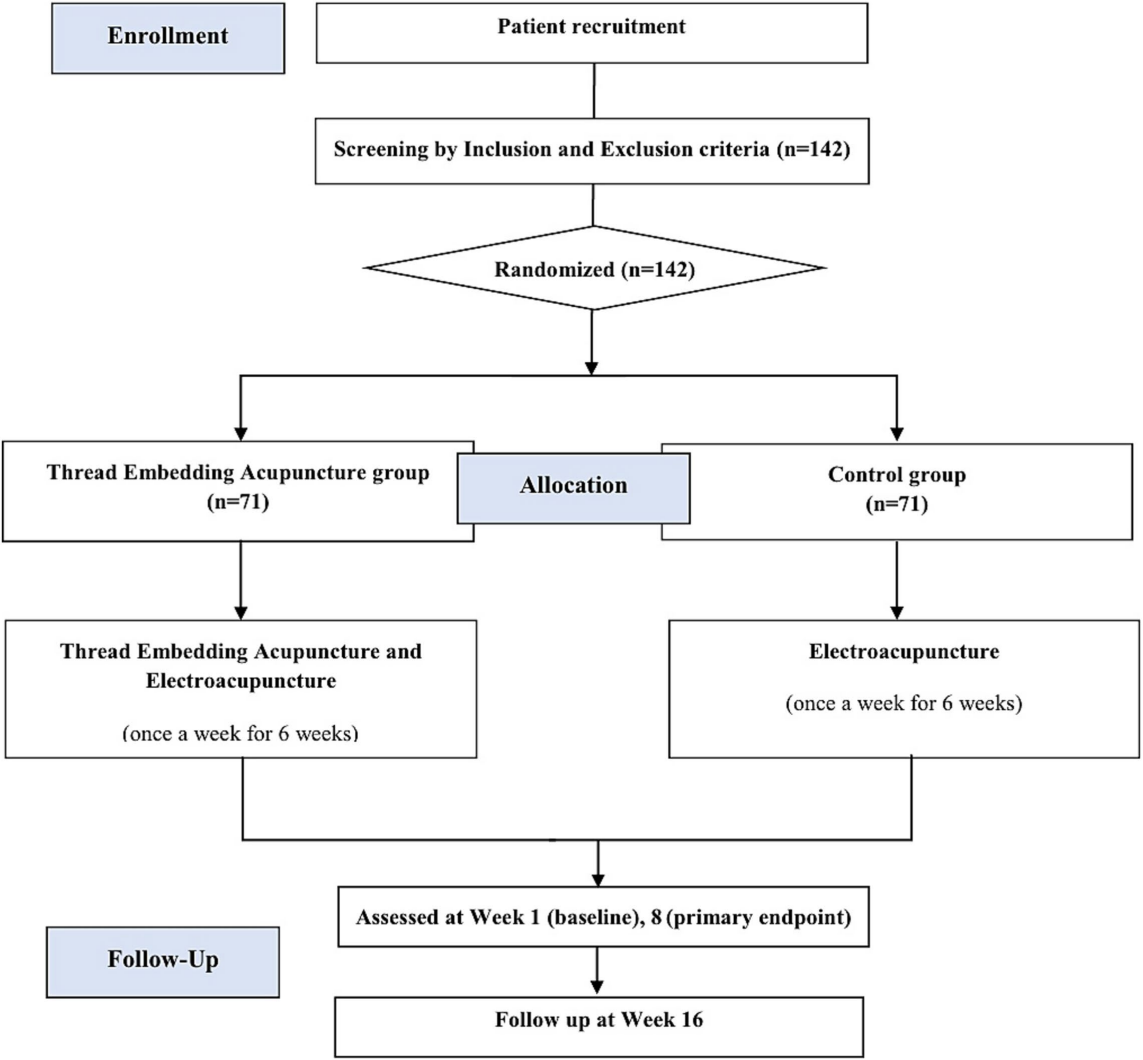
Flow chart of the study.

This study will be a multi-center, randomized, assessor-blinded clinical trial with a single treatment (TEA). A total of 142 participants with FNP sequelae more than 12 months after onset will be recruited from the Kyung Hee University Oriental Medicine Hospital at Gangdong and the Pusan National University Korean Medicine Hospital. Detailed information regarding the study will be provided to all potential participants beforehand, and only those who agree to participate and sign an informed consent form will undergo eligibility screening. Those who meet the inclusion and exclusion criteria will be randomly assigned to the TEA or control groups. The study will consist of a screening period, six treatment sessions (Week 1–6) over 6 weeks and two additional follow-up sessions (Week 8 and 16), and will be conducted according to the designated scheduled appointment. The trial design is summarized in Figure 1. Table 1 shows more details regarding the SPIRIT schedule for enrollment, interventions, and assessments. The enrollment is expected to begin on June 22, 2023 and end on July 31, 2025. Data collection is scheduled to be completed by November 30, 2025, with preliminary conclusions expected to be drawn by December 31, 2025.

**Table 1:** SPIRIT schedule of the study.

Timepoint	Study period					
	Screening	Allocation and enrollment	Post-allocation		Follow-up	
		Week 1 (Baseline)	Week 2–5	Week 6	Week 8 (Primary endpoint)	Week 16
Visit		Visit 1	Visit 2–5	Visit 6	Visit 7	Visit 8
Visit window			±3	±3	±3	±7
Enrollment						
Informed consent	O					
Eligibility screening	O					
Randomization		O				
Intervention						
Intervention group: TEA + EA		O	O	O		
Control group: EA		O	O	O		
Assessments						
Sunnybrook facial grading system	O	O		O	O	O
Facial disability index^*^		O		O	O	O
Numeric rating scale for stiffness		O		O	O	O
Synkinesis assessment questionnaire		O		O	O	O
Patient global impression of change				O	O	O
Euro-Qol 5-dimension 5-level		O		O	O	O
Economic cost evaluation		O		O	O	O
Adverse event		O	O	O	O	O

* Primary outcome.

SPIRIT, standard protocol items: recommendations for interventional trials; TEA, thread embedding acupuncture; EA, electroacupuncture.

### 2.2 Participants

Applicants will evaluate each volunteer based on the admission and exclusion criteria. The assigned plan of each group will be explained in full to the volunteers by the research assistants, along with the study procedure and consent documents. After signing an informed consent form and accepting the study invitation, qualified individuals schedule a baseline evaluation.

#### 2.2.1 Inclusion criteria

Participants will be included if they meet the following criteria: (1) Adults aged between 19 and 80 years old, (2) Peripheral facial palsy more than 12 months after onset, (3) 80 points or less in a total score by the Sunnybrook Facial Grading System, (4) Synkinesis in more than one facial region by the Sunnybrook Facial Grading System, (5) Not receiving any treatment for the peripheral facial palsy sequelae within the last month, (6) Voluntary participation with written consent after hearing an explanation of the clinical trial.

#### 2.2.2 Exclusion criteria

Participants will be excluded if they meet the following criteria: (1) Thread embedding acupuncture or botox treatment on the face within the last 3 months, (2) Hypersensitivity to electroacupuncture or thread embedding acupuncture, metal allergy, severe keloid skin, and other skin sensitivities, (3) Bilateral peripheral facial palsy sequelae, (4) Facial paralysis due to cerebrovascular disease, (5) Intracranial tumor around the facial nerve that was not removed, (6) Skin diseases or blood clotting disorders for which thread embedding acupuncture is contraindicated, (7) Pregnant or planning to become pregnant, (8) Mental illness or inability to communicate in compliance with clinical trials, (9) Other reasons judged unsuitable by the researcher.

### 2.3 Criteria for withdrawal and termination

In accordance with ethical principles protecting patient autonomy, participants will have the right to withdraw from the study at any time and for any reason. The study may be discontinued in cases of consent withdrawal by the participant, the presence of a medical condition that endangers the participant’s safety, or the investigator’s determination that further participation is inappropriate for other reasons. I n the event of a severe adverse reaction, the investigator will permanently discontinue the clinical trial for the affected participant.

### 2.4 Sample size calculation

The level of significance (*α*) is set at 0.05 (two-sided test). Type II error (*β*) is set at 0.2, with a test power of 80%. No clinical trial of thread embedding with the same design could be found through a literature review. Therefore, the sample size was calculated by applying the mean difference of 9.29 and the standard deviation of 17.146 for the Facial Disability Index’s Physical score as the primary efficacy evaluation indicator, based on the results of previously conducted, similar clinical trials on the sequelae of FNP (10).

Considering a dropout rate of 20% (11), 71 participants per group will be needed. A total of 142 participants will be assigned to the study: 71 persons per group, with 102 patients (51 in the TEA group, 51 in the control group) at the one medical center and 40 patients (20 in the TEA group, 20 in the control group) at the other medical center. If an institution’s recruitment ends early, additional participants will be recruited competitively to ensure a smooth enrollment process.

### 2.5 Randomization

Participants who voluntarily sign the informed consent form will be assessed for their eligibility to participate in the clinical trial based on the inclusion and exclusion criteria. The final selected participants will be assigned to their respective groups using a random allocation table provided by two clinical trial institutes. According to the random allocation details, identification codes will be assigned to participants in the order they were recruited, and treatment will be implemented in each group accordingly. An independent researcher, not affiliated with the main clinical trial team, will employ Excel 2019 (Microsoft Corporation, Redmond, WA, USA) to conduct block randomization. This method will feature a block random assignment and the concealment of block sizes to prevent those responsible for participant recruitment and group allocation from knowing the block sizes (12).

### 2.6 Allocation concealment

The random allocation table will be managed by a third researcher not involved in the intervention and evaluation, and the trial personnel, based on the random allocation table, will randomly assign participants by opening the sequentially numbered, opaque envelopes provided by an independent envelope administrator, not related to the study. The random assignment will be predetermined and cannot be changed once carried out. As it is not feasible to blind the interventionists and participants in this study design, evaluator blinding will be implemented. The evaluator who will not participate in the intervention will be blinded to group allocation.

### 2.7 Intervention

In the TEA and control group, six electroacupuncture sessions will be performed once a week for 6 weeks. In the TEA group, after completing the acupuncture treatment, the embedding acupuncture treatment will be carried out once a week for 6 weeks for six sessions. During the trial period (6 weeks), no other interventions will be performed apart from the designated treatments. Electroacupuncture and TEA therapies will be performed by a specialist in acupuncture or a specialist trainee under the supervision of a specialist. The interventionists will complete an educational program through a trial supervisor-led workshop to ensure that treatments are conducted according to the established methods in all studies.

#### 2.7.1 TEA

**Figure 2:**
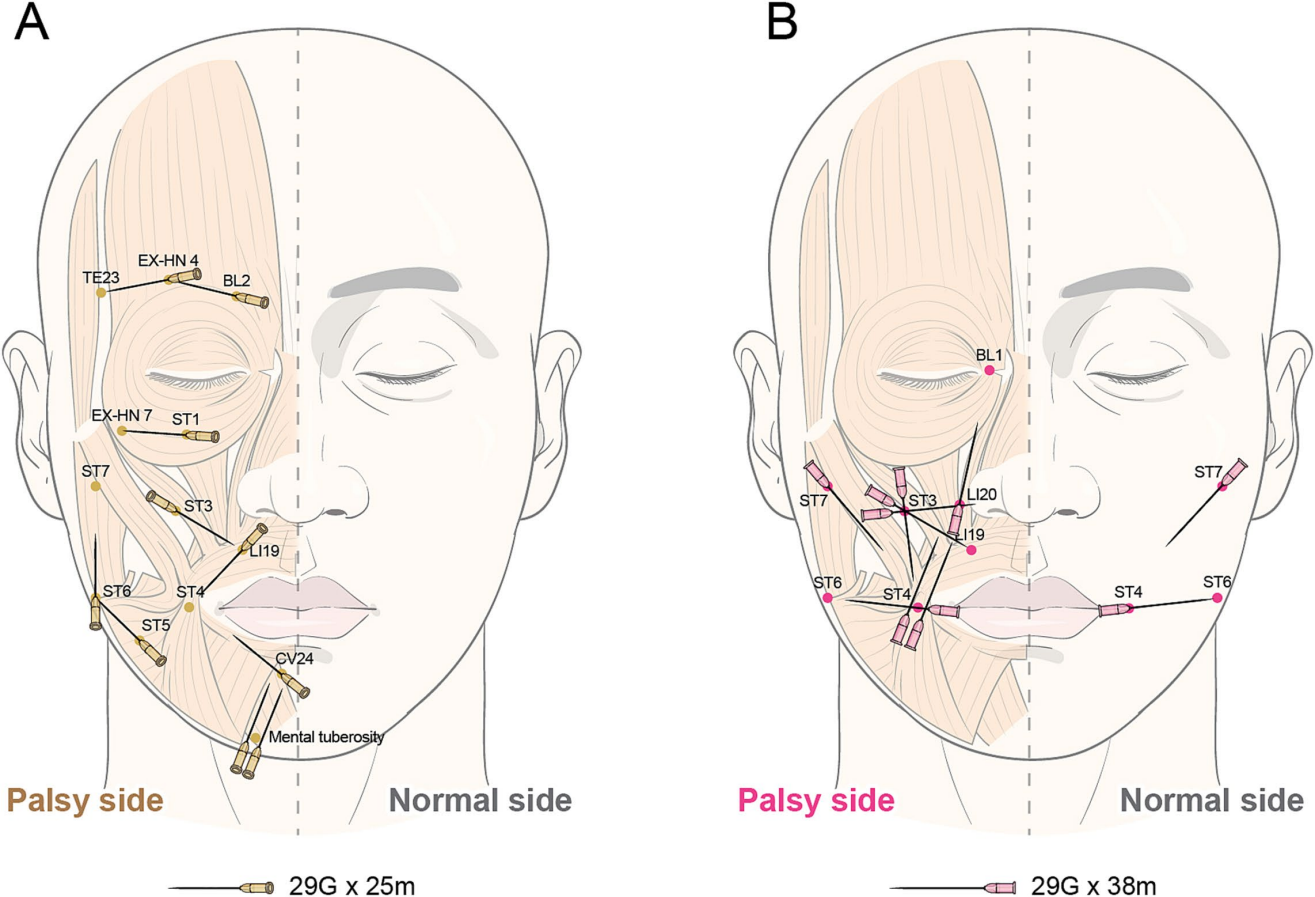
Insertion location of thread embedding acupuncture.

TEA will be administered using a 29 G, 25-mm and 38-mm TEA needle (Hyundae Meditech, Wonju, South Korea) on 20 predefined acupoints selected by an expert group of Korean medical doctors according to STRICTA (Table 2 and Figure 2).

**Table 2:** Details of the TEA using the STRICTA 2010 checklist.

Item	Details	
1. Acupuncture rationale	1a. Style of acupuncture	TEA
	1b. Reasoning for treatment provided	By the consensus of a group of clinical experts, modified from that used in a previous study.
	1c. Extent to which treatment was varied	No variation
2. Details of needling	2a. Number of needle insertions per subject per session	20 acupoints
	2b and 2c. Names of points used and depth of insertion, based on a specified unit of measurement or on a particular tissue level	with 29G × 25 mm (affected side): BL2 to EX-HN 4, EX-HN 4 to TE23, ST1 to EX-HN 7, ST3 to LI19, LI19 to ST4, ST4 to ST6, ST6 to ST7, CV24 to ST4, mental tuberosity to CV24. with 29G × 38 mm (affected side): ST4 to LI20, LI20 to BL1, ST7 to ST4, ST4 to ST6, SI18 to LI20, SI18 to LI19, SI18 to ST4. with 29G × 38 mm (unaffected side): ST4 to ST6, ST7 to ST4.
	2d. Response sought	Thread embedding can produce sensations similar to acupuncture, such as soreness, distension, or heaviness, but the reactions tend to be more pronounced due to the thicker and longer threads used in the procedure.
	2e. Needle stimulation	Thread embedding
	2f. Needle retention time	No retention time
	2g. Needle type	TEA (29G × 25 mm and 38 mm)
3. Treatment regimen	3a. Number of treatment sessions	6 sessions
	3b. Frequency and duration of treatment sessions	Once a week for 6 weeks
4. Other components of treatment	4a. Details of other interventions administered to the acupuncture group	Electroacupuncture
	4b. Setting and context of treatment, including instructions to practitioners, and information and explanations to patients	Minimized conversation between practitioner and patient.
5. Practitioner background	5a. Description of participating acupuncturists	Specialists of the acupuncture and moxibustion department with at least 3 years of clinical experience.
6. Control and comparator intervention	6a. Rationale for the control or comparator in the context of the research question, with sources that justify this choice	Electroacupuncture will be used as a comparator, in this manner, the specific therapeutic effect of TEA will only be displayed.
	6b. Precise description of the control or comparator	0.20 × 30 mm sized acupuncture will be used within 5–20 mm depth. After the administration of acupuncture, electro-stimulation will be applied for 20 min.

TEA, thread embedding acupuncture; STRICTA, standards for reporting interventions in clinical trials of acupuncture.

#### 2.7.2 Electroacupuncture

Sixteen needles of 0.20 × 30 mm size will be used for administration at the following acupoints at a depth of 5–20 mm. The acupoint locations were determined according to the standard acupoints guidelines provided by the Korean Medicine Convergence Research Information Center (13).

Twelve affected side acupoints are BL2, TE23, GB14, ST3, TE17, ST4, ST6, GV26, CV24, ST7, ST 18 and ST3. Two bilateral acupoints are LI4 and LR3. After the insertion, electrical stimulation will be applied for 20 min at a frequency of 4 Hz, inducing muscle contraction with an intensity that does not cause discomfort to the participants. Stimulated acupoints are BL2 to TE23, ST4 to ST6, SI18 to ST3.

### 2.8 Outcome measurement

#### 2.8.1 Facial Disability Index

The primary outcome measurement is the physical function score with Facial Disability Index. The Facial Disability Index is the patient-reported evaluation questionnaire that consists of a total of 10 items, which are further divided into five items related to physical function (Physical score) and five items related to social/well-being function (Social-Wellbeing score). Based on the scores of individual items, the physical function (Physical score) is calculated out of 100 points, and the social/well-being function (Social-Wellbeing score) is also calculated out of 100 points. With a total of 200 points being the maximum score, a lower score indicates a more severe functional impairment due to facial disorders (14).

#### 2.8.2 Sunnybrook facial grading system

The evaluator assesses facial movement due to FNP and derives a final score within the maximum range of 100 points based on scores for 3 Resting symmetry items, scores for 5 Symmetry of Voluntary Movement items, and scores for 5 Synkinesis items (15).

#### 2.8.3 Numeric rating scale for stiffness

The discomfort from stiffness experienced in four areas—the forehead, around the eyes, cheeks, and mouth—is evaluated using a numeric rating scale of 0–10 points.

#### 2.8.4 Synkinesis assessment questionnaire

The total score will be calculated by evaluating the nine typical symptoms of associated movements on a scale of 1–5 points. Divide the total score by 45 and multiply by 100 to evaluate the score out of 100 points. A higher score indicates a more severe level of associated movements as evaluated by the patient.

#### 2.8.5 Patient global impression of change

This method allows research participants to evaluate the overall subjective improvement of symptoms on a 7-point scale, ranging from “much improved” to “much worse.”

#### 2.8.6 Euro-Qol 5-dimension 5-level

The EuroQol-5 Dimension (EQ-5D-5L) is a widely used indirect measurement method for calculating the quality weight of a specific health state using preference scores assigned to function levels after assessing the health state from various angles. The EQ-5D-5L consists of five items, each asking about the degree of mobility, self-care, usual activities, pain/discomfort, and anxiety/depression (16). An element of the EQ-5D, a standardized tool created by the EuroQol Group for gauging general health state, is the Visual Analogue Scale (VAS) for the EQ-5D. A vertical visual analogue scale with endpoints labeled “Best imaginable health state” at 100 and “Worst imaginable health state” at 0 is used in the VAS to quantify an individual’s self-rated health. This gives a precise assessment of the patient’s perceived health status when the test is finished (17).

#### 2.8.7 Economic cost evaluation

Both formal and informal healthcare costs will be surveyed. Formal healthcare costs will collect information on the type of medical institution, the number of visits (number of visits, number of admissions, total number of hospital days), and total out-of-pocket expenses, distinguishing between outpatient and inpatient treatment. Informal healthcare costs will investigate the types and expenditure amounts for over-the-counter medications, health-functional foods, medical devices, massages, and other items.

#### 2.8.8 Adverse events

Adverse reactions will be collected through research participants’ symptom reporting and researchers’ observations and will be identified by the frequency of occurrence between groups. Researchers will evaluate the causal relationship between each treatment method and the adverse effects experienced using a 6-point scale (18). (1 = related, 2 = probably related, 3 = possibly related, 4 = probably not related, 5 = definitely not related, and 6 = unknown) Moreover, all adverse effects will be classified into three levels according to Spilker’s classification method (19). (Mild (1): No treatment is required, and the participant’s daily life (function) is not significantly impaired; Moderate (2): The participant’s daily life (function) is significantly impaired, and treatment may be needed, but the condition improves after treatment; Severe (3): Severe adverse reactions require extensive treatment and may leave sequelae.)

### 2.9 Statistical analysis

Statistical analysis will be performed using the Full Analysis Set (FAS) analysis group, which includes participants who have received treatment at least once, and missing values will be addressed using multiple imputations. All statistical analyses will be based on two-sided tests, with a significance level set at 5%. The SAS version 9.1.3 statistical package (SAS Institute, Cary, NC, USA) will be used as the analytical tool. Descriptive statistics will be provided for each group concerning the demographic characteristics and baseline clinical data of the participants. Comparisons between groups will be conducted using the independent t-test, the Wilcoxon rank-sum test for continuous data, and the Chi-square test or Fisher’s Exact test for categorical data. For the analysis of secondary efficacy evaluation variables in Weeks 6 & 16 (secondary endpoint), the same method as the primary efficacy evaluation variables will be used. The paired t-test or Wilcoxon signed-rank test will also be employed to analyze the differences in pre- and post-treatment measurements within each group. The repeated measured Analysis of Variance (repeated measures ANOVA) will be used to assess the interaction between time and group over time.

### 2.10 Data collection and management

Participants and clinical trial information will be documented in the electronic medical record (EMR) system of the medical institution. Data collection, management procedures, and information on blinding will be provided only to designated researchers who will handle and crosscheck the trial data. Evaluation records completed by patients and assessors will be kept confidential and maintained as supporting evidence, recorded in both the EMR and paper, and subsequently entered into an electronic case report form (eCRF) provided by MyTrial (Bethesda Soft Co., Ltd., Seoul, Korea). Researchers will be assigned role-based permissions to access, modify, and input data within the eCRF system, with all activities logged for accountability.

### 2.11 Quality control

The study procedures and documentation will be periodically monitored according to the Korean Good Clinical Practice guidelines.

## 3 Discussion

In this multi-center, randomized controlled trial protocol, we aim to evaluate the effectiveness and safety of TEA alongside electroacupuncture for FNP. Adhering to the SPIRIT and STRICTA guidelines, our methodologically rigorous study design will extend previous research by utilizing a larger, diverse cohort, meticulous inclusion and exclusion criteria, and a comprehensive range of primary and secondary outcome measures.

To overcome the limitations of a previous clinical trial and design a trial applicable to clinical practice, we conducted a preliminary survey targeting Korean medical doctors with clinical experience in performing TEA for FNP. The survey revealed that 29-gauge mono-needles were most commonly employed to mitigate contracture between the lips and nasolabial folds. TEA session numbers averaged 5.9 per patient, with few instances surpassing 20 needles (20). Considering these usage trends, we established TEA administration sites, intervals, and quantities.

This study will use electroacupuncture as the control group to restore paralyzed nerves and muscles (21). According to reviews of earlier animal studies, electroacupuncture treatment favors the expression of elements involved in nerve regeneration (22–24). The results of the aforementioned survey showed that roughly 60 practitioners are using TEA treatment along with electroacupuncture (20), which has been clinically confirmed through systematic literature reviews (25, 26). Electroacupuncture, with these effects, is employed in clinical settings to treat acute symptoms and sequelae of facial paralysis along with acupuncture or TEA.

Beyond its role in FNP, acupuncture intervention holds significant importance across various diseases, particularly in neurological and brain cognitive disorders. Recent advanced works have highlighted the profound modulatory effects of acupuncture on neural diseases and brain cognition, employing a range of sophisticated measurements to evaluate its effectiveness. For instance, studies have demonstrated neural activation via acupuncture in patients with major depressive disorder, as evidenced by functional near-infrared spectroscopy (27). Furthermore, acupuncture’s efficacy in modulating brain activity has been evaluated using periodic-aperiodic EEG measurements (28), and its impact on spectral power and functional connectivity in the human brain has been meticulously investigated (29). Acupuncture therapy has also shown promising effects on patients with mild cognitive impairment, as observed through functional near-infrared spectroscopy (30). The development of neural manifold decoders for acupuncture stimulations, employing representation learning, further underscores the intricate interaction between acupuncture and the brain (31). These findings collectively suggest that acupuncture serves as a clinically important intervention across a spectrum of diseases, extending far beyond localized conditions.

The potential mechanisms by which acupuncture, and specifically the combined TEA and electroacupuncture treatment in our protocol, act on FNP are multifaceted. Electroacupuncture, as supported by existing literature, promotes the expression of neurotrophic factors such as Nerve Growth Factor (NGF), Ciliary Neurotrophic Factor (CNTF), and Glial Cell-Derived Neurotrophic Factor (GDNF) (21), thereby facilitating the regeneration of damaged facial nerves. It also improves Schwann cell morphology and supports neuronal survival and growth, contributing to the recovery of paralyzed muscles and the alleviation of sequelae such as synkinesis (22–24). Thread Embedding Acupuncture, on the other hand, involves the subcutaneous insertion of absorbable threads that provide continuous physical and biochemical stimulation. This leads to improved local circulation, enhanced collagen formation, and modulation of inflammation, all of which contribute to tissue regeneration (32–34). The synergy between electroacupuncture and TEA in our protocol aims to create an optimal biochemical environment for nerve regeneration, induce electrical activity in paretic muscles, and leverage unique acupuncture-specific responses, such as the “Deqi” sensation, to modulate central and peripheral nervous systems, thereby maximizing therapeutic outcomes.

This randomized controlled trial is designed to delve deeper into the implications of this study protocol. While acute FNP treatments are often well-standardized, long-term sequelae such as synkinesis, contracture, spasm, and crocodile tears syndrome present a significant clinical challenge with limited and often insufficiently evidenced treatment options, including nerve transplantation or botulinum toxin injections. To assess whether the sequelae have been resolved, it is crucial to consider both the perception of physical changes and self-recognized improvements in functional areas (35). In this study, we will conduct a comprehensive evaluation using the physician-rated Sunnybrook Facial Grading System (SFGS), as well as patient self-assessments via the Facial Disability Index (FDI) and Numeric Rating Scale (NRS).

The upcoming randomized controlled trial aims to examine the effectiveness and safety of TEA combined with electroacupuncture in managing FNP. This study, designed to address previous research limitations, will use rigorous methodologies and an adequate sample size. The objective is to explore TEA’s potential benefits, disadvantages, and possible adverse events to uncover improved therapeutic strategies.

The potential applications of the findings from this study will extend significantly into clinical practice and future research. If proven effective and safe, TEA could offer a new, minimally invasive therapeutic option for patients suffering from long-term FNP sequelae. Furthermore, the successful validation of TEA in this context could stimulate broader research into its applications for various other health conditions. For instance, TEA could be explored as a rehabilitation treatment for post-stroke hemiplegia or other neurological motor disorders, chronic musculoskeletal pain syndromes, or neuropathic pain management, leveraging its continuous stimulatory effects. In the cosmetic field, it could complement existing facial aesthetic acupuncture techniques to enhance skin elasticity, reduce wrinkles, and correct facial asymmetry. This study, therefore, not only aims to establish evidence-based standards for TEA in treating FNP but also serves as a crucial foundation for strengthening the scientific validation of complementary and alternative medicine, thereby expanding our understanding and utilization of TEA for a diverse range of health concerns.
